# The psychological impact of COVID-19 pandemic on healthcare professionals: A cross-sectional study

**DOI:** 10.1192/j.eurpsy.2022.1279

**Published:** 2022-09-01

**Authors:** M. Vouros, P. Koutoukoglou, E. Jelastopulu

**Affiliations:** 1 424 General Military Training Hospital, Psychiatric Dept., Thessaloniki, Greece; 2 424 General Military Training Hospital, Oncology Dept., Thessaloniki, Greece; 3 University of Patras, School of Medicine, Dept. Of Public Health, Patras, Greece

**Keywords:** Healthcare professionals, Demographic characteristics, psychological distress, Covid-19

## Abstract

**Introduction:**

The COVID-19 pandemic was immediately realized to pose a considerable threat both to the physical, and the mental health of people. For healthcare professionals, it marks frantic work rhythms, anxiety for their patients and exposure to an invisible enemy. Those who hold administrative positions are called upon to make unprecedented decisions, facing a high degree of uncertainty. Hence, hospital staff is expected to experience severe psychological distress.

**Objectives:**

The aim of this study was to investigate the psychological distress and possible associations with demographic characteristics, professional duties, hierarchy and predisposing factors to severe COVID-19 disease.

**Methods:**

Online questionnaires were distributed to all employees of two hospitals in Thessaloniki, Greece, from March until May 2021. The questionnaires comprised two sections, one concerning the aforementioned purported risk factors, and another involving three psychometric scales, i.e. Coronavirus Anxiety Scale, Coronavirus Reassurance-Seeking Behaviors Scale and Obsession with COVID-19 Scale.

**Results:**

The psychological pressure experienced by healthcare professionals was low, compared to the literature. A history of COVID-19 disease, existence of predisposing factors to severe COVID-19 illness and frequent contact with infected patients were shown to significantly increase the likelihood of psychological distress. Furthermore, an age of 30-34 years, a higher level of education, existence of infected family members and non-vaccination were identified as possible risk factors.

**Conclusions:**

Contrary to previous research results, our sample did not experience severe COVID-19-related psychological distress. Nevertheless, emphasis should be placed on initiatives to support the mental health of this professional group, as many of them do struggle with psychological difficulties.

**Disclosure:**

No significant relationships.

